# Ubiquitin-specific peptidase 25 ameliorates hepatic steatosis by stabilizing peroxisome proliferator-activated receptor alpha

**DOI:** 10.1016/j.jbc.2024.107876

**Published:** 2024-10-11

**Authors:** Peihao Liu, Xin Song, Qingxia Chen, Li Cen, Chenxi Tang, Chaohui Yu, Chengfu Xu

**Affiliations:** 1 Department of Gastroenterology, The First Affiliated Hospital, Zhejiang University School of Medicine, Hangzhou, China; 2 Department of Gastroenterology, Affiliated Hangzhou First People’s Hospital, Westlake University School of Medicine, Hangzhou, China; 3 Key Laboratory of Integrated Traditional Chinese and Western Medicine for Biliary and Pancreatic Diseases of Zhejiang Province, Hangzhou, China; 4 Hangzhou Hospital & Institute of Digestive Diseases, Hangzhou, China

**Keywords:** ubiquitin-specific peptidase 25, hepatic steatosis, peroxisome proliferator-activated receptor alpha, nonalcoholic fatty liver disease, deubiquitination

## Abstract

Nonalcoholic fatty liver disease (NAFLD) is the most common chronic liver disease worldwide. Ubiquitin-specific peptidase 25 (USP25) in adipocytes has been proven to be involved in insulin resistance, a noteworthy characteristic of NAFLD. However, the roles of USP25 in NAFLD remain unclear. In this study, we aimed to elucidate the role of USP25 in NAFLD. Hepatic USP25 protein levels were measured in NAFLD patients and models. USP25 expression was manipulated in both mice and cells to evaluate its role in NAFLD. A downstream target of USP25 in NAFLD progression was identified through proteomic profiling analyses and confirmed. Additionally, a USP25 inhibitor was used to determine whether USP25 could be a viable treatment target for NAFLD. We found that USP25 protein levels were significantly decreased in the livers of NAFLD patients and NAFLD model mice. USP25 protein levels were also decreased in both mouse primary hepatocytes and Huh7 cells treated with free fatty acids (FFAs). We also found that Usp25 knockout mice presented much more severe hepatic steatosis when they were fed a high-fat diet. Similarly, knocking down USP25 in Huh7 cell lines aggravated FFA-induced steatosis, whereas USP25 overexpression ameliorated FFA-induced steatosis in Huh7 cell lines. Further proteomic profiling revealed that the peroxisome proliferator-activated receptor alpha (PPARα) signaling pathway was a downstream target of USP25, which was confirmed in both mice and cell lines. Moreover, USP25 could stabilize PPARα by promoting its deubiquitination. Finally, a USP25 inhibitor exacerbated diet-induced steatosis in mice. In conclusion, USP25 may play a role in NAFLD through the PPARα signaling pathway and could be a potential therapeutic target for NAFLD.

Nonalcoholic fatty liver disease (NAFLD) represents a spectrum of liver conditions ranging from simple steatosis to nonalcoholic steatohepatitis, which includes inflammation and fibrosis (1). NAFLD is the most prevalent chronic liver disease worldwide, affecting approximately a quarter of the population (2). It is closely associated with metabolic syndromes (3) such as obesity, insulin resistance (IR), type 2 diabetes, and dyslipidemia and can lead to liver cirrhosis or even hepatocellular carcinoma (4). The pathogenesis of NAFLD is complex and not fully understood and involves a combination of environmental, genetic, and lifestyle factors (1), which contributes to the current lack of Food and Drug Administration–approved drugs for its treatment. The high prevalence and potential severity of NAFLD underscore the critical need for understanding its underlying mechanisms and developing effective treatments.

Ubiquitin-specific peptidase 25 (USP25), first identified by Valero *et al*. as the product of a gene at 21q11.2 (5), is a deubiquitination enzyme that plays a crucial role in the regulation of protein degradation *via* the ubiquitin‒proteasome system (6, 7), a pathway essential for maintaining cellular homeostasis. USP25 has been shown to be involved in various pathophysiological processes, such as the immune response (8, 9, 10), cancer development (11, 12, 13), and inflammatory pathways (9, 14, 15). Additionally, recent research has highlighted the potential role of USP25 in IR (16, 17), a noteworthy feature of NAFLD. Specifically, USP25 can regulate glucose transporter type 4 (GLUT4) translocation in adipocytes, thereby influencing the insulin response. Despite the critical role of IR in NAFLD, there is no research on the relationship between USP25 and NAFLD.

Here, we found that USP25 deficiency markedly exacerbated the development of diet-induced hepatic steatosis in mice by directly binding to peroxisome proliferator-activated receptor alpha (PPARα) and decreasing its K48-linked deubiquitination, leading to reduced beta-oxidation of lipids in hepatocytes. More importantly, a USP25 inhibitor also exacerbated diet-induced hepatic steatosis in mice, which could unveil novel therapeutic targets and strategies for managing this increasingly prevalent liver disease.

## Results

### The protein expression level of USP25 was markedly reduced in both NAFLD patients and NAFLD models

**Figure 1:**
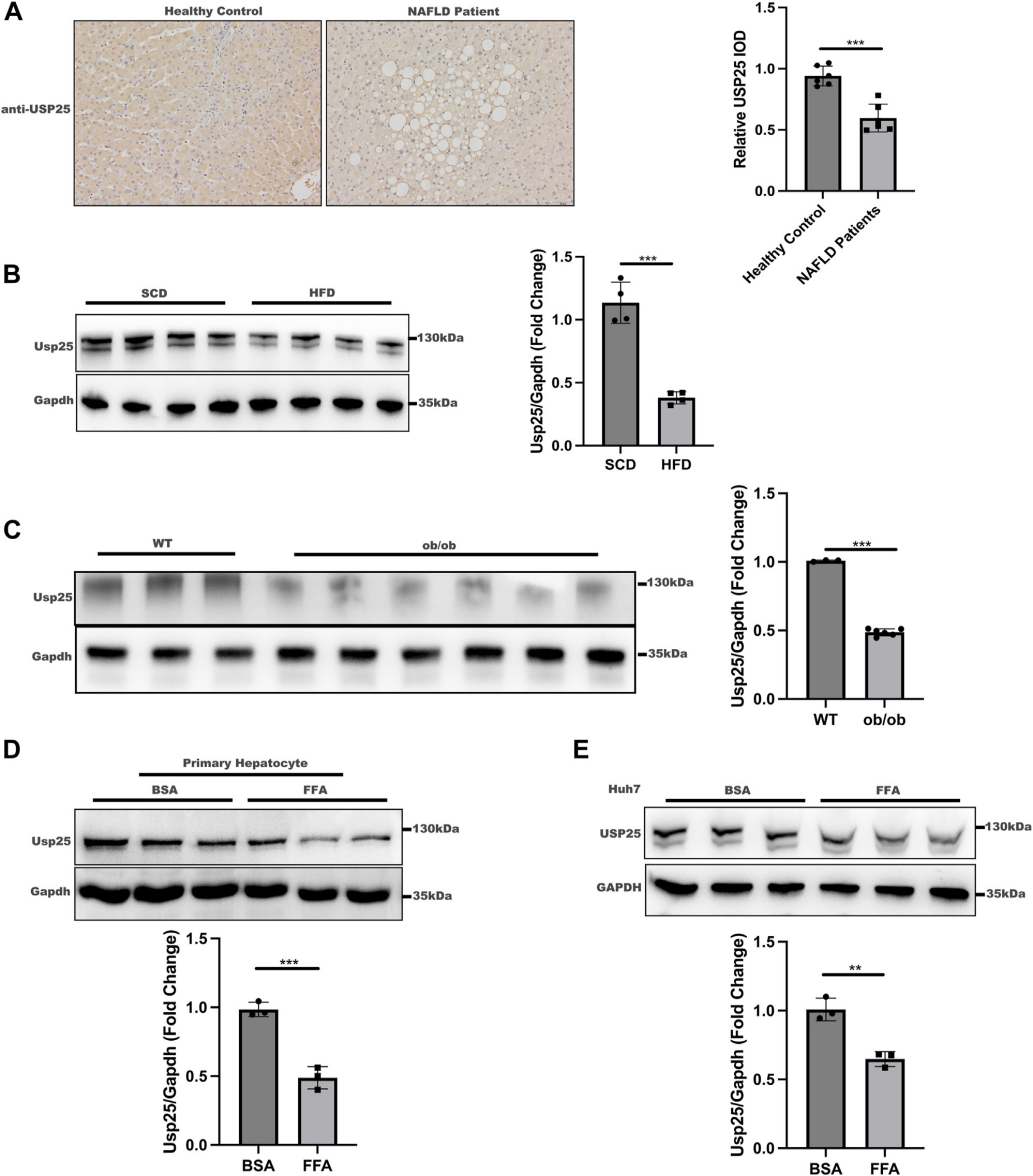
**USP25 expression is markedly downregulated in NAFLD.***A*, *left panel*: representative IHC staining of USP25 in the livers of healthy controls and NAFLD patients (*n* = 6 per group, 200 × magnification). *Right panel*: statistical analysis of USP25 expression in healthy controls and NAFLD patients. *B*, *left panel*: representative Western blot showing Usp25 in the livers of mice fed a HFD (*n* = 4) or SCD (*n* = 4) for 16 weeks. *Right panel*: quantification of hepatic Usp25 protein levels normalized to Gapdh levels. *C*, *left panel*: representative Western blot showing Usp25 protein expression in the livers of 6- to 8-week-old male wildtype (WT) mice (*n* = 3) and ob/ob mice (*n* = 6) fed a SCD. *Right panel*: quantification of hepatic Usp25 protein levels normalized to Gapdh levels. *D*, *upper panel*: representative Western blot showing Usp25 in primary hepatocytes from 6- to 8-week-old male WT mice treated with BSA or FFA for 24 h. *Lower panel*: quantification of Usp25 protein levels normalized to Gapdh. *E*, *upper panel*: representative Western blot of Usp25 in Huh7 cells treated with BSA or FFA for 24 h. *Lower panel*: quantification of Usp25 protein levels normalized to Gapdh. The data are expressed as the mean ± standard deviation (SD) and were analyzed by Student's *t* test. ∗∗*p* < 0.01; ∗∗∗*p* < 0.001. BSA, bovine serum albumin; FFA, free fatty acid; HFD, high-fat diet; IHC, Immunohistochemical; IOD, integrated optical density; NAFLD, nonalcoholic fatty liver disease; SCD, standard chow diet; USP25, ubiquitin-specific peptidase 25.

To investigate the specific roles of USP25 in NAFLD, we first assessed its expression levels. USP25 protein expression was significantly lower in the livers of NAFLD patients than in those of healthy controls, as depicted in Fig. 1*A*, which was consistent with USP mRNA expression levels reported previously (18). Additionally, we observed a significant decrease in Usp25 protein levels in the livers of high-fat diet (HFD)-induced NAFLD mice compared with those in the livers of mice fed a standard chow diet (SCD) (Fig. 1*B*). Similarly, Usp25 protein levels were markedly lower in the livers of ob/ob mice than in those of their wildtype (WT) counterparts (Fig. 1*C*). Intriguingly, when primary mouse hepatocytes were exposed to free fatty acids (FFAs) to induce steatosis, Usp25 protein levels were significantly lower than those in hepatocytes treated with bovine serum albumin (Fig. 1*D*). Consistent with the findings in primary hepatocytes, USP25 protein levels were notably lower in FFA-treated Huh7 cell lines than in bovine serum albumin–treated controls (Fig. 1*E*). These results suggest that USP25 expression in hepatocytes is disrupted under NAFLD conditions.

### Knockout of USP25 aggravated hepatic steatosis in NAFLD models

**Figure 2:**
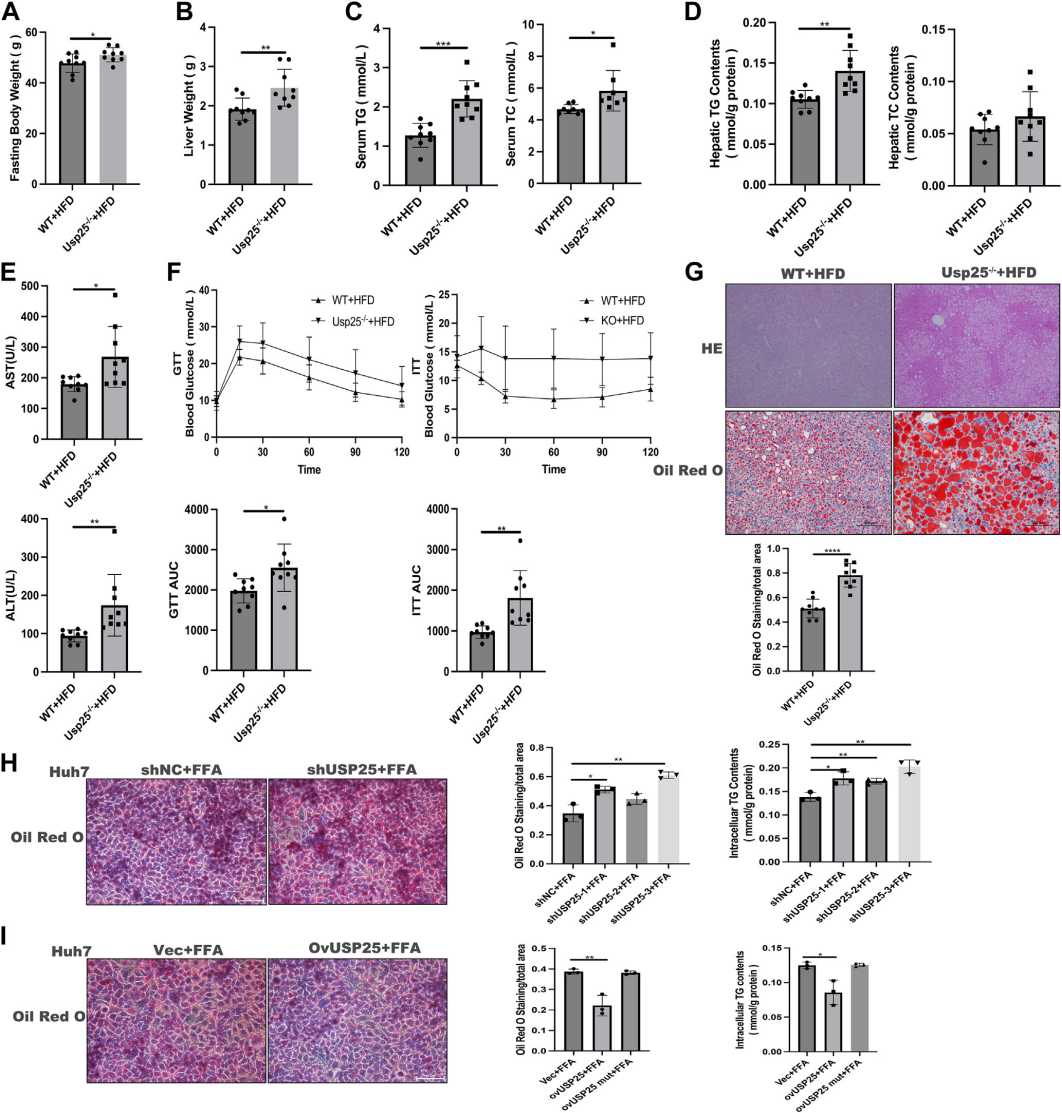
**Usp25 deficiency exacerbated HFD-induced hepatic steatosis.***A*, fasting body weights of wildtype (WT) and Usp25 knockout (Usp25^−/−^) mice fed a HFD for 16 weeks (*n* = 9 in each group). *B*, liver weights of WT and Usp25^−/−^ mice fed a HFD for 16 weeks (*n* = 9 in each group). *C*, serum TG and TC levels in WT and Usp25^−/−^ mice fed a HFD for 16 weeks (*n* = 9 in each group). *D*, hepatic TG and TC levels in WT and Usp25^−/−^ mice fed a HFD for 16 weeks (*n* = 9 in each group). *E*, serum ALT and AST levels in WT and Usp25^−/−^ mice fed a HFD for 16 weeks (*n* = 9 in each group). *F*, GTTs and ITTs of the indicated mice. *G*, *lower panel*: representative H&E staining and oil red O staining of the indicated groups (200 × magnification). *Upper panel*: statistical analysis of oil red O staining. *H*, *left panel*: oil red O staining of the indicated groups (*n* = 3 in each group, 200 × magnification). *Middle panel*: statistical analysis of oil red O staining. *Right panel*: cellular TG contents of the indicated groups. *I*, *left panel*: Oil red O staining of the indicated groups (*n* = 3 in each group, 200 × magnification). *Middle panel*: statistical analysis of oil red O staining. *Right panel*: Cellular TG contents of the indicated groups. The data are expressed as the means ± standard deviations (SDs), and (*A*–*G*) were analyzed by Student's *t* test. *H*–*I*, the data were analyzed by one-way ANOVA. ∗*p* < 0.05; ∗∗*p* < 0.01; ∗∗∗*p* < 0.001; ∗∗∗∗*p* < 0.0001. ALT, aminotransferase; AST, aspartate aminotransferase; AUC, area under the curve; FFA, free fatty acid; GTT, glucose tolerance test; HFD, high-fat diet; ITT, insulin tolerance test; TC, total cholesterol; TG, triglyceride; USP25, ubiquitin-specific peptidase 25.

To investigate the role of USP25 in NAFLD progression, Usp25 knockout (Usp25^−/−^) mice were generated and fed a HFD to induce NAFLD, with WT littermates serving as controls. The Usp25 protein was effectively depleted, as evidenced by immunoblotting (Fig. S2). After 16 weeks of HFD feeding, the body and liver weights of the Usp25^−/−^ mice were greater than those of the WT controls (Fig. 2, *A* and *B*), whereas there was no significant difference in body or liver weight between the WT mice and Usp25^−/−^ mice fed the SCD (Fig. S2). Compared with WT control mice, Usp25^−/−^ mice also presented significantly greater serum triglyceride (TG) and total cholesterol (TC) levels (Fig. 2*C*) and greater intrahepatic TG content (Fig. 2*D*). Aminotransferase and aspartate aminotransferase levels were also greater in Usp25^−/−^ mice than in WT control mice fed a HFD (Fig. 2*E*). Furthermore, Usp25 deficiency exacerbated HFD-induced IR, as measured by the insulin tolerance test (ITT) and glucose tolerance test (GTT) (Fig. 2*F*). Histological staining with hematoxylin and eosin and Oil Red O confirmed greater lipid accumulation in the livers of Usp25^−/−^ mice fed a HFD (Fig. 2*G*) but not those fed a SCD (Fig. S2). Additionally, manipulating USP25 expression (Fig. S2) in Huh7 cell lines *via* specific shRNA or overexpression plasmids revealed that USP25 knockdown aggravated FFA-induced lipid accumulation (Fig. 2*H*), whereas USP25 overexpression ameliorated FFA-induced lipid accumulation (Fig. 2*I*). These results collectively indicate that USP25 expression influences hepatic steatosis both *in vivo* and *in vitro*.

### Usp25 participated in NAFLD via the PPARα signaling pathway in mice

**Figure 3:**
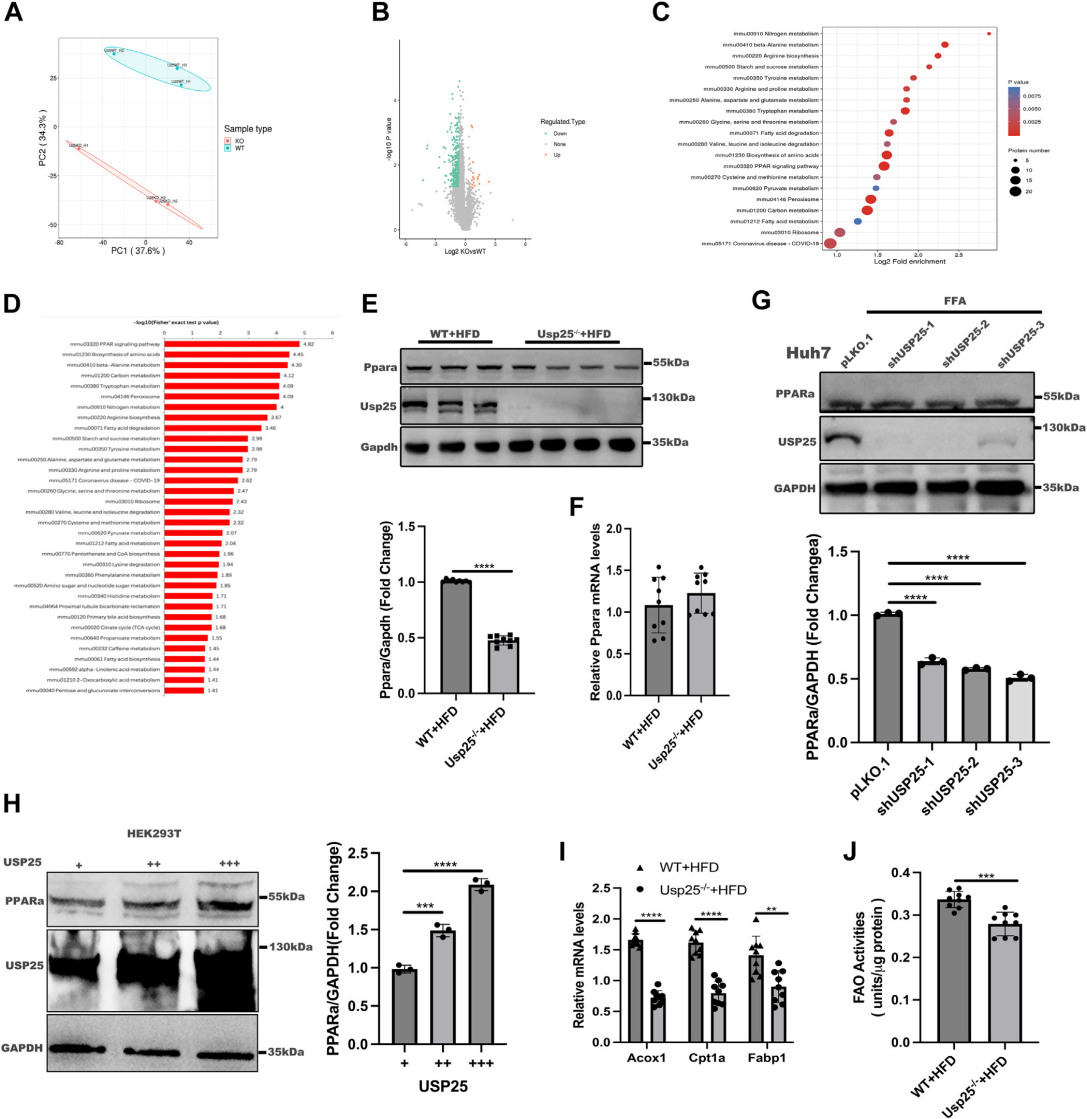
**Usp25 could modulate Pparα expression.***A*, principal component analysis (PCA) of each indicated group (KO, Usp25 knockout). *B*, downregulated and upregulated proteins in the indicated groups. *C*, KEGG pathway analysis. *D*, Fisher’s exact test for KEGG pathway analysis. *E*, *upper panel*: representative Western blot showing Usp25 and PPARα protein expression in the indicated groups (*n* = 9 in each group). *Bottom panel*: quantification of the PPARα protein level normalized to that of Gapdh. *F*, relative Pparα mRNA expression in the indicated groups (*n* = 9 in each group). *G*, *upper panel*: representative Western blot showing USP25 and PPARα protein expression in the indicated groups (*n* = 3 in each group). *Bottom panel*: quantification of the PPARα protein level normalized to that of GAPDH. *H*, *left panel*: representative Western blots showing USP25 and PPARα protein expression in the indicated groups (*n* = 3 in each group). *Right panel*: quantification of the PPARα protein level normalized to that of GAPDH. *I*, relative mRNA expression of the indicated genes in the indicated groups (*n* = 9 in each group). *J*, fatty acid oxidation (FAO) activities were measured in the indicated groups (*n* = 9 in each group). The values are expressed as the means ± standard deviations (SDs). *E*, *F*, *I*, *J*, differences were analyzed by Student's *t* test. (*G*–*H*) were analyzed by one-way ANOVA. ∗∗*p* < 0.01; ∗∗∗*p* < 0.001; ∗∗∗∗*p* < 0.0001. Acox1, acyl-Coenzyme A oxidase 1; Cpt1α, carnitine palmitoyltransferase 1α; Fabp1, fatty acid binding protein 1; KEGG, Kyoto Encyclopedia of Genes and Genomes; PPARα, peroxisome proliferator-activated receptor alpha; USP25, ubiquitin-specific peptidase 25; WT, wildtype.

To investigate the mechanisms through which USP25 regulates NAFLD, we conducted ubiquitin proteomic analyses on liver samples from HFD-fed Usp25^−/−^ mice and their WT counterparts, as depicted in Fig. 3*A*. Our ubiquitin proteomic profiling revealed that 270 proteins were upregulated, and 13 proteins were downregulated (Fig. 3*B*). We subsequently employed Kyoto Encyclopedia of Genes and Genomes (KEGG) analysis to characterize the biological pathways affected by these proteins and identified significant enrichment of Ppar signaling pathways (Fig. 3, *C* and *D*). Specifically, KEGG pathway analysis highlighted alterations in the Pparα signaling pathway (Fig. S3).

We further evaluated Pparα protein expression levels in the livers of HFD-fed Usp25 KO mice and WT controls. Consistent with our proteomic findings, Pparα protein levels were significantly lower in the livers of HFD-fed Usp25^−/−^ mice than in those of their WT littermates (Fig. 3*E*), whereas Pparα mRNA expression did not significantly change (Fig. 3*F*). To explore this further, we utilized shRNA to establish stable USP25-knockdown Huh7 cell lines, which were subsequently treated with FFA. As depicted in Fig. 3*G*, PPARα protein levels were significantly decreased in USP25-knockdown Huh7 cells following FFA treatment. Additionally, we observed a correlation between USP25 and PPARα protein levels (Fig. 3*H*), indicating the potential role of USP25 in modulating PPARα expression in hepatic steatosis. Notably, the mRNA levels of downstream targets of Pparα (acyl-Coenzyme A oxidase 1, carnitine palmitoyltransferase 1α, and fatty acid binding protein 1) were also significantly reduced (Fig. 3*I*), suggesting that USP25 may influence hepatic steatosis through PPARα regulation, given the pivotal role of Pparα in lipid beta-oxidation (19). Moreover, we found that the target protein levels of Pparα (carnitine palmitoyltransferase 1α and acyl-Coenzyme A oxidase 1) were significantly reduced, as shown in Fig. S4. We also found that fatty acid oxidation activity was significantly lower in the livers of HFD-fed Usp25^−/−^ mice than in those of their WT littermates (Fig. 3*J*).

### USP25 interacts with PPARα and stabilizes it through deubiquitination

**Figure 4:**
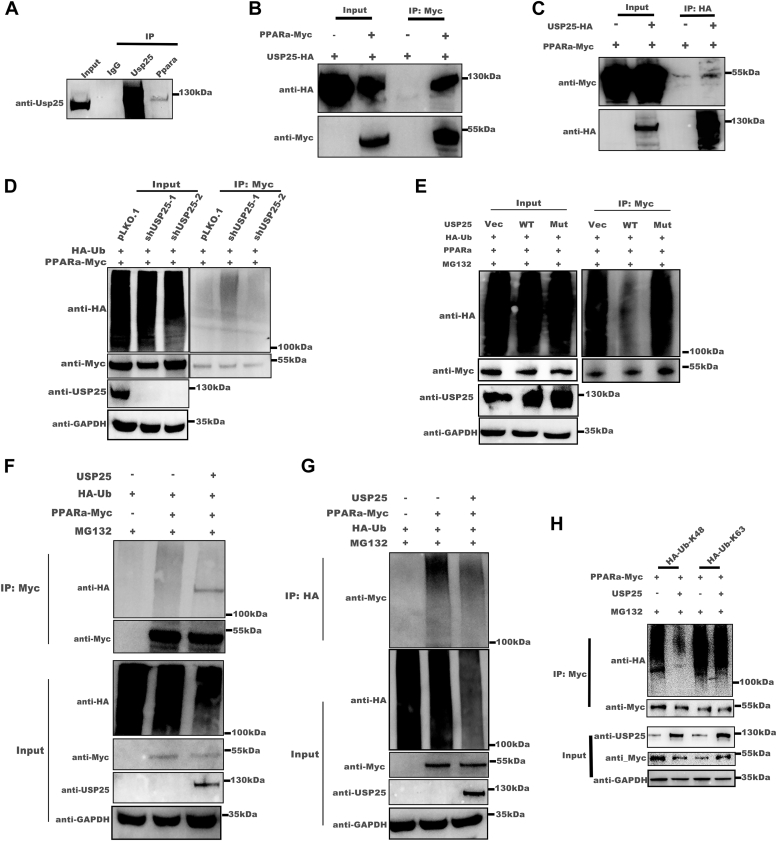
**USP25 interacts with PPARα and stabilizes it through deubiquitination.***A*, endogenous co-IP assays were conducted to evaluate the interaction between Usp25 and PPARα in the livers of HFD-fed mice. *B* and *C*, exogenous co-IP assays were conducted to evaluate the interaction between USP25 and PPARα in HEK293T cells transfected with HA-tagged USP25 and a Myc-tagged PPARα plasmid. *D*, Western blotting analysis of the ubiquitination of PPARα in the stable USP25-knockdown Huh7 cell line MG132 was performed for 6 h before harvesting. *E*, Western blot analysis of PPARα ubiquitination in Huh7 cells transfected with USP25 wildtype or mutant (C178S) plasmids. MG132 was added for 6 h before harvest. *F* and *G*, Western blot analysis of PPARα ubiquitination in HEK293T cells transfected with the indicated plasmids. MG132 was added for 6 h before harvest. *H*, Western blotting analysis of the ubiquitination of PPARα in HEK293T cells transfected with mutant ubiquitin (K48, K63) and PPARα. MG132 was added for 6 h before harvest. HFD, high-fat diet; TC, total cholesterol; TG, triglyceride; PPARα, peroxisome proliferator-activated receptor alpha; USP25, ubiquitin-specific peptidase 25.

To further investigate how USP25 regulates PPARα expression, we first examined the interaction between the two proteins. Coimmunoprecipitation experiments demonstrated that endogenous Usp25 interacts with PPARα in the mouse liver (Fig. 4*A*). Subsequent *in vitro* studies confirmed a direct interaction between USP25 and PPARα (Fig. 4, *B* and *C*). Given the role of USP25 as a deubiquitinating enzyme, we conducted ubiquitin assays and revealed that overexpression of USP25 reduced the level of ubiquitinated PPARα, whereas USP25 knockdown increased the level of ubiquitinated PPARα (Fig. 4, *D*–*G*). Importantly, we observed that USP25 specifically deubiquitinates K48-linked ubiquitin chains on the PPARα protein (Figs. 4*H*, S5). Given the important role of K48-linked ubiquitin in protein degradation (20), these results suggest that USP25 might stabilize PPARα through K48-linked deubiquitination.

### Overexpressing Pparα in the livers of Usp25^−/−^ mice ameliorated hepatic steatosis in diet-induced NAFLD

**Figure 5:**
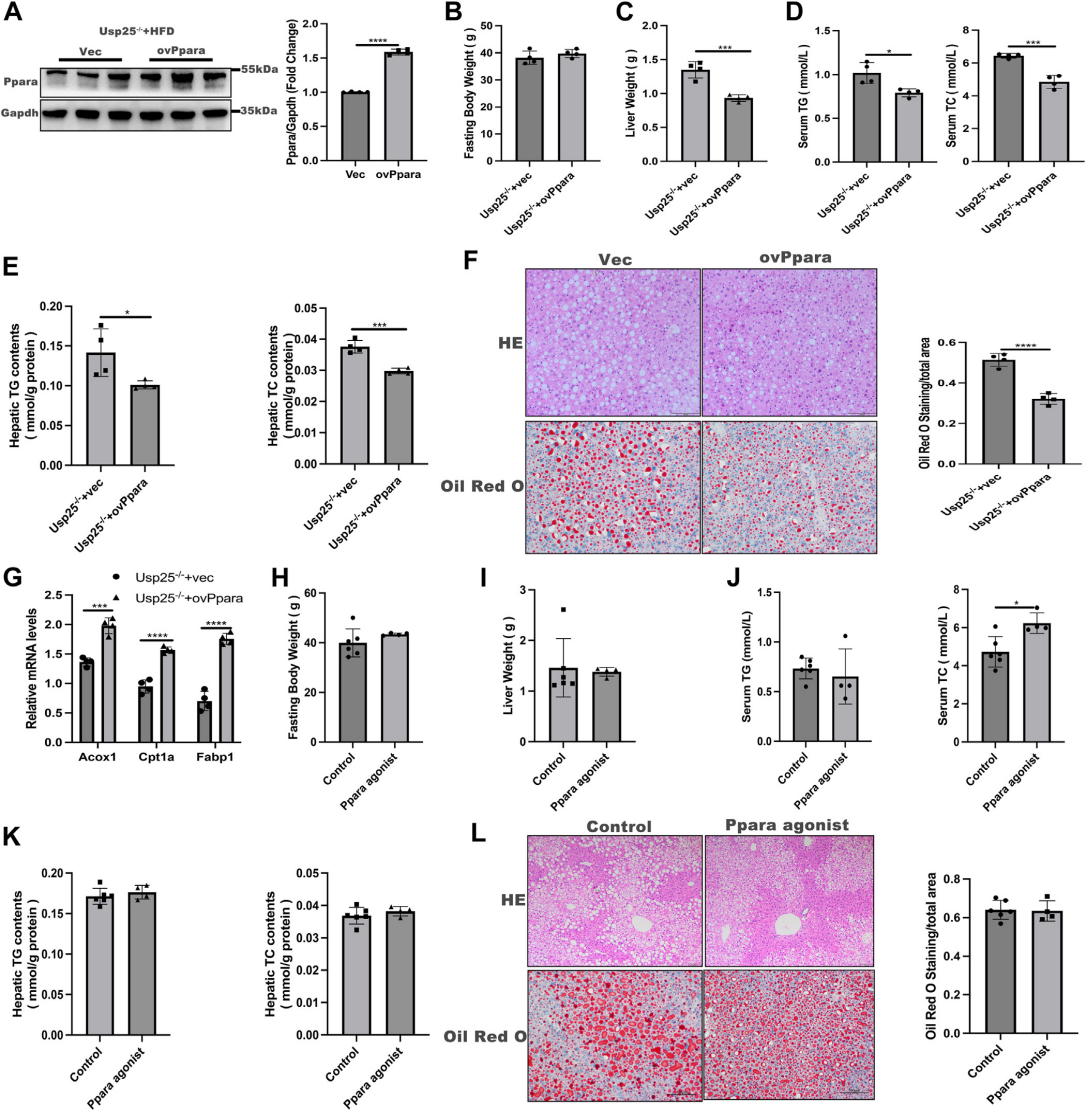
**Overexpressing Pparα in the livers of Usp25**^**−/−**^**mice ameliorated hepatic steatosis in diet-induced NAFLD.***A*, representative Western blot (*left panel*) of PPARα protein in the livers of the indicated groups (*n* = 4 in each group); quantification of PPARα protein levels (*right panel*) normalized to Gapdh. Fasting body weight (*B*) and liver weight (*C*) of the indicated groups (*n* = 4 in each group). Serum TG and TC levels (*D*) and hepatic TG and TC contents (*E*) of the indicated groups (*n* = 4 in each group). (*F*) Representative H&E staining and oil red O staining of the indicated groups (*left panel*, *n* = 4 in each group; 200 × magnification). Statistical analysis of oil red O staining (*right panel*). *G*, relative mRNA expression of the indicated genes in the indicated groups (*n* = 4 in each group). Fasting body weight (*H*) and liver weight (*I*) of the indicated groups (*n* = 6 in the control group, *n* = 4 in the PPARα agonist group). Serum TG and TC levels (*J*) and hepatic TG and TC levels (*K*) in the indicated groups (*n* = 6 in the control group, *n* = 4 in the PPARα agonist group). *L*, representative H&E staining and oil red O staining of the indicated groups (*left panel*, *n* = 6 in the control group, *n* = 4 in the PPARα agonist group; 200 × magnification). Statistical analysis of oil red O staining (*right panel*). The values are expressed as the mean ± standard deviation (SD) and were analyzed by Student's *t* test. ∗*p* < 0.05; ∗∗∗*p* < 0.001; ∗∗∗∗*p* < 0.0001. NAFLD, nonalcoholic fatty liver disease; PPARα, peroxisome proliferator-activated receptor alpha; TC, total cholesterol; TG, triglyceride; USP25, ubiquitin-specific peptidase 25.

To assess whether overexpressing Pparα in Usp25^−/−^ mice could mitigate NAFLD *in vivo*, we administered an adeno-associated virus carrying Pparα into the tail vein to increase hepatic Pparα expression in Usp25^−/−^ mice. Pparα protein levels were significantly increased, as depicted in Fig. 5*A*. While the overexpression of PPARα did not affect body weight (Fig. 5*B*), liver weight was notably lower in the Pparα-overexpressing group than in the vector control group (Fig. 5*C*). Furthermore, hepatic Pparα overexpression led to significant reductions in the serum and intrahepatic TG and TC levels in HFD-fed Usp25^−/−^ mice (Fig. 5, *D* and *E*). Histological analyses confirmed that hepatic Pparα overexpression significantly ameliorated hepatic steatosis in these mice. Moreover, Pparα overexpression upregulated the expression of downstream targets (Fig. 5*G*). In contrast, treatment with a Pparα agonist in HFD-fed USP25 KO mice did not alleviate metabolic phenotypes (Fig. 5, *H*–*K*) or hepatic steatosis (Fig. 5*L*), as confirmed by histological analysis, likely due to decreased Pparα levels. These findings underscore the role of Usp25 in NAFLD through the regulation of Pparα expression in mice.

### Inhibition of Usp25 exacerbated hepatic steatosis in HFD-fed mice

**Figure 6:**
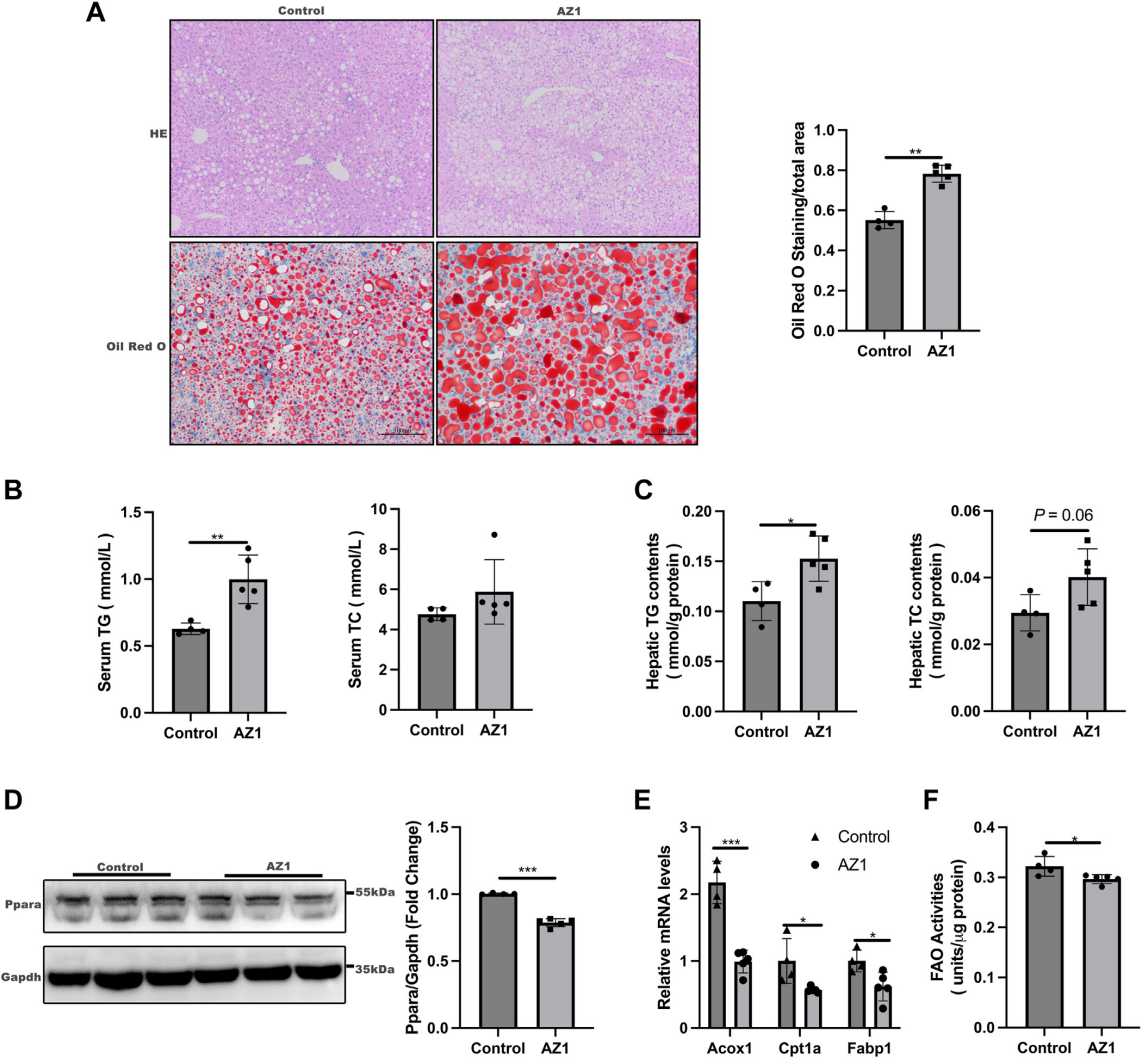
**The Usp25 inhibitor exacerbated hepatic steatosis in HFD-fed mice.***A*, representative H&E staining and oil red O staining of the indicated groups (*left panel*, *n* = 4 in the control group, *n* = 5 in the AZ1 group; 200 × magnification). Statistical analysis of oil red O staining (*right panel*). *B* and *C*, serum TG and TC levels (*B*) and hepatic TG and TC levels (*C*) in the indicated groups (*n* = 4 in the control group, *n* = 5 in the AZ1 group). *D*, *left panel*: Representative Western blot showing PPARα in the livers of the indicated groups of mice (*n* = 4 in the control group, *n* = 5 in the AZ1 group). *Right panel*: Quantification of the PPARα protein level normalized to that of Gapdh. *E*, relative mRNA expression of the indicated genes in the indicated groups (*n* = 4 in the control group, *n* = 5 in the AZ1 group). *F*, fatty acid oxidation (FAO) activities were measured in the indicated groups (*n* = 4 in the control group, *n* = 5 in the AZ1 group). The values are expressed as the mean ± standard deviation (SD) and were analyzed by Student's *t* test. ∗*p* < 0.05; ∗∗*p* < 0.01; ∗∗∗*p* < 0.001. HFD, high-fat diet; PPARα, peroxisome proliferator-activated receptor alpha; TC, total cholesterol; TG, triglyceride; USP25, ubiquitin-specific peptidase 25.

To further investigate Usp25 as a potential target for NAFLD treatment, we administered the Usp25 inhibitor AZ1 to mice fed a HFD. After 12 weeks of HFD feeding, the WT mice were randomly divided into two groups and treated with either water or AZ1 for 10 days. Histological analysis confirmed that AZ1 significantly exacerbated hepatic steatosis (Fig. 6*A*). Additionally, compared with the control treatment, AZ1 treatment markedly increased the serum and intrahepatic TG levels (Fig. 6, *B* and *C*). Furthermore, hepatic PPARα protein levels were significantly reduced in AZ1-treated mice (Fig. 6*D*), as was the expression of downstream PPARα targets (Figs. 6*E*, S4). In addition, fatty acid oxidation activity was significantly lower in the livers of AZ1-treated mice than in those of their control littermates (Fig. 6*J*). These findings suggest that Usp25 may indeed be a promising therapeutic target for NAFLD.

## Discussion

In this study, we identified novel regulatory roles and mechanisms of USP25 in NAFLD. USP25 expression was markedly decreased in both human and mouse livers during NAFLD progression. Deletion of USP25 significantly exacerbated NAFLD in mice, whereas overexpression of USP25 in cell lines significantly inhibited lipid accumulation. Through enrichment analysis of differentially expressed proteins, we identified PPARα as a downstream target of USP25. The binding of PPARα to USP25 was confirmed, and USP25 facilitated K48-linked deubiquitination, thereby inhibiting the proteasomal degradation of PPARα. Furthermore, the inhibition of USP25 exacerbated NAFLD, indicating that USP25 could be a promising therapeutic target for NAFLD.

Dysregulation of the hepatic ubiquitination–deubiquitination balance has been detected in NAFLD progression (21), indicating its crucial role in NAFLD development and progression. Several deubiquitination enzymes have been proven to be involved in different stages of hepatic steatosis, affecting lipid accumulation, ROS, and inflammation signaling pathways in NAFLD (22, 23). For example, members of the USP family, such as USP11 (24), USP14 (25), USP15 (26) and USP18 (27), have been identified as crucial players in NAFLD progression. However, the exact role of USP25, another member of the USP family, remains unclear. Gawrieh *et al*. reported that USP25 mRNA expression is significantly downregulated in NAFLD (18), which might subsequently affect lipid metabolism. Additionally, USP25 was identified as a protease required for insulin-stimulated TUG cleavage and GLUT4 translocation in adipocytes, which could impact IR. Sadler *et al*. reported that USP25 binds to tankyrase and regulates trafficking of the facilitative glucose transporter GLUT4 in adipocytes (16, 17). In our study, we found a significant decrease in USP25 protein levels during NAFLD development in both human and mouse models. More importantly, the deletion of USP25 exacerbated hepatic steatosis in a diet-induced NAFLD mouse model and in FFA-induced cell steatosis, which indicated that USP25 participated in NAFLD progression.

In the present study, we identified PPARα as a substrate of USP25 *via* proteomic profiling. PPARα, a key regulator of lipid metabolism, regulates the peroxisomal beta-oxidation pathway of fatty acids and is involved in the progression of NAFLD (19). Considering that PPARα can be ubiquitinated and degraded (28) and that USP25 is a deubiquitination enzyme, we hypothesized that USP25 regulates NAFLD by modulating the deubiquitination and stability of PPARα. Indeed, our results indicated that USP25 impaired the K48-linked ubiquitination and degradation of PPARα and that resupplying PPARα in USP25-deficient mice ameliorated diet-induced hepatic steatosis. However, in this study, we found that a PPARα agonist could not ameliorate diet-induced hepatic steatosis in Usp25^−/−^ mice, which was inconsistent with the findings of previous studies showing that a PPARα agonist could ameliorate diet-induced hepatic steatosis in wildtype mice (29, 30). On the one hand, a PPARα agonist was administered to Usp25^−/−^ mice fed a HFD, which presented much more severe hepatosteatosis and might resist the anti-obesogenic effects of the PPARα agonist. On the other hand, a lower level of PPARα expression and more severe hepatosteatosis in Usp25^−/−^ mice fed a HFD might counteract the anti-obesogenic effects of PPARα agonists. Additionally, we found that a USP25 inhibitor could exacerbate diet-induced hepatic steatosis. Therefore, considering that it is impossible to cure all NAFLD patients with a single target, we speculate that the combination of a USP25 agonist and a PPARα agonist may be a promising strategy for NAFLD therapy.

There are several limitations to this study. First, the regulatory mechanisms of USP25 in NAFLD are not fully understood. Previous studies have shown that the type I interferon-IRF7 axis can mediate the transcription of the USP25 gene after viral infection or lipopolysaccharide treatment (31) and that miRNA-200c can reduce both the messenger RNA and protein levels of USP25 in non-small cell lung cancer (32). Future studies are needed to elucidate the regulatory mechanisms of USP25 in NAFLD. Second, systemic Usp25 knockout mice, which can potentially be influenced by other organs, were used in this study. However, the effects of Usp25 on hepatic steatosis were replicated in primary hepatocytes and liver cell lines. Liver-specific Usp25 knockout mice will be used in future studies to address this issue. Finally, the potential therapeutic value of a USP25 agonist in NAFLD patients needs to be evaluated in future research.

In conclusion, our study identified USP25 as a critical positive regulator of NAFLD. USP25 facilitates lipid oxidation by binding to PPARα and inhibiting its K48-linked ubiquitination-mediated degradation. These findings suggest that USP25 could be a promising therapeutic target for NAFLD.

## Experimental procedures

### Patients

Liver biopsies from healthy controls and NAFLD patients were obtained from liver transplant donors or patients undergoing liver surgeries at the First Affiliated Hospital, Zhejiang University School of Medicine. The inclusion criteria were based on the presence of hepatic steatosis (NAFLD patients) or the absence of hepatic steatosis (healthy controls), with no other discernible causes, such as alcohol abuse or viral hepatitis. All procedures that involved human sample in this study were consistent with the principles outlined in the Declaration of Helsinki and were approved by Ethics Committee of the First Affiliated Hospital, Zhejiang University School of Medicine.

### Mice and treatments

Usp25 knockout mice (Usp25^−/−^), which were generated *via* CRISPR/Cas9-mediated genome engineering, were purchased from Cyagen Biosciences, Inc. Specifically, exon 2 and exon 3 of the Usp25 gene were targeted, as shown in Fig. S1. Usp25^−/−^ mice and their wildtype littermates were housed at the Zhejiang Academy of Medical Science under a 12-h light/dark cycle in a specific pathogen-free environment with *ad libitum* access to food and water. Male ob/ob mice were purchased from the Model Animal Research Center of Nanjing University. The NAFLD model was established by feeding mice a HFD (Research Diet) for 12 to 16 weeks, while mice fed a SCD served as controls. To overexpress hepatic PPARα, AAV9-TBG-PPARα was injected through the tail vein at a dose of 5 × 10^11^ genome copies per mouse 1 week prior to establishing the NAFLD model. The PPARα agonist Wy-14643 (MCE) (100 mg per kg body weight per day) was administered daily by gavage for 4 weeks after 12 weeks of HFD feeding, with corn oil serving as the control. The USP25 inhibitor AZ1 (MCE) (40 mg per kg body weight per day) was administered by gavage for 9 days after 16 weeks of HFD feeding, with corn oil used as the control. All animal experiments were performed according to guidelines approved by the Animal Care and Use Committee of the First Affiliated Hospital, Zhejiang University School of Medicine.

### Cell culture and treatments

Primary hepatocytes from the mice were isolated as previously described. Briefly, a male mouse was anesthetized, and the liver was perfused and digested with D-Hank’s solution (Genom) and collagenase type IV (Gibco), respectively. The digested liver suspension was collected and centrifuged, and the precipitate containing primary hepatocytes was incubated at 37 °C in a 5% CO_2_ incubator. The HEK293T and Huh7 cell lines were purchased from the Chinese Academy of Science and cultured in Dulbecco's modified Eagle’s medium supplemented with 10% (vol/vol) fetal bovine serum and 1% (vol/vol) penicillin/streptomycin. The authenticity of the cell lines were validated using STR profiling, and all cell lines were free from *mycoplasma* contamination. All the cells were cultured at 37 °C in a 5% CO2 incubator. FFA, a mixture of palmitic acid and oleic acid at a final concentration of 1 mM, was administered to cells for 24 h to establish a cellular model of steatosis. Huh7 and HEK293T cells were transfected with the indicated plasmids purchased from Generay *via* PEI Max (Polysciences) according to the manufacturer’s instructions. The sequences of shRNA for USP25 are provided in Table S2.

### Histological analyses

Hematoxylin and eosin staining and oil red O staining were used to analyze liver or cell pathology. Immunohistochemical staining was used to evaluate the expression level of USP25 in human liver sections. All the sections were imaged at 200 × magnification (Olympus).

### Metabolic analysis and measurement of biochemical indices

For the GTT, the mice were fasted for 16 h and then injected intraperitoneally with 1 mg/g glucose (Sigma Aldrich) solution. For the ITT, the mice were fasted for 6 h and then injected intraperitoneally with 0.75 U/kg insulin. For both the GTT and ITT, blood glucose concentrations were determined at baseline and at 15, 30, 60, 90, and 120 min after injection in tail blood *via* a glucometer (LifeScan). TG, TC, aminotransferase, and aspartate aminotransferase levels were assayed with the indicated commercial kits (Nanjing Jiancheng Bioengineering) according to the manufacturers’ instructions. Fatty acid oxidation activities were measured with the indicated commercial kit (AssayGenie).

### LC‒MS/MS analysis

The enriched tryptic peptides were dissolved in solvent A and directly loaded onto a homemade reversed-phase analytical column (25 cm in length, 100 μm i.d.). The mobile phase consisted of solvent A (0.1% formic acid and 2% acetonitrile/in water) and solvent B (0.1% formic acid in acetonitrile). Peptides were separated with the following gradient at a constant flow rate of 450 nl/min on a NanoElute UHPLC system (Bruker Daltonics): 0 to 18 min, 6 to 22% B; 18 to 22 min, 22 to 30% B; 22 to 26 min, 30 to 80% B; and 26 to 30 min, 80% B. The peptides were subjected to capillary source chromatography followed by mass spectrometry on a timsTOF Pro mass spectrometer. The electrospray voltage applied was 1.7 kV. The precursors and fragments were analyzed with a TOF detector. The timsTOF Pro was operated in data-independent parallel accumulation serial fragmentation mode. The full MS scan was set at 100 to 1700 m/z (MS/MS scan range), and 10PASEF (MS/MS mode)-MS/MS scans were acquired per cycle. The MS/MS scan range was set at 400 to 1200 m/z, and the isolation window was set at 25 m/z. The differentially expressed proteins were subjected to KEGG enrichment analysis.

### Western blotting

Total protein from tissues and cells was extracted with RIPA buffer (Applygen) supplemented with proteinase and phosphatase inhibitors (Sigma). Protein concentrations were quantified with a BCA protein assay kit (Applygen). Equal amounts of total protein were separated *via* 10% sodium dodecyl sulfate‒polyacrylamide gel electrophoresis and then transferred to polyvinylidene fluoride membranes (Millipore). Next, the membranes were blocked in 5% (wt/vol) nonfat milk and sequentially incubated with specific primary antibodies overnight and with an HRP-labeled secondary antibody for another hour. Finally, the signals were detected with an enhanced chemiluminescence kit (Applygen) and visualized with an imaging system. The antibodies used in this study are listed in Table S1. The protein bands were quantitatively analyzed by Image J software.

### RNA extraction and qPCR analysis

Total RNA was extracted from tissues and cells *via* RNA plus (Takara), after which the quality and quantification of the RNA were evaluated. cDNA libraries were constructed *via* a One Step PrimeScriptTM RT‒PCR Kit (Takara), and sequential quantitative PCR (qPCR) was performed on an ABI Prism 7500 sequence detection system (Applied Biosystems) with SYBR Green (Takara). Relative quantification was performed *via* the ΔΔCt method. The sequences of the primers used in this study are provided in Table S2.

### *In vivo* ubiquitin assays

Tissues and cells were harvested and lysed with SDS lysis buffer (50 mM Tris–HCl pH 7.4, 150 mM NaCl, 1 mM EDTA, and 1% SDS) and then denatured by heating at 95 °C for 10 min. The denatured supernatants were subsequently diluted with SDS-free buffer (50 mM Tris–HCl pH 7.4, 150 mM NaCl, 1 mM EDTA, and 1% Triton X-100) until the final concentration of SDS was 0.1%. Ubiquitin levels were then detected by Western blotting with the indicated antibodies.

### Coimmunoprecipitation

Tissues and cells transfected with the indicated plasmids were harvested and lysed in IP lysis buffer (Thermo Fisher), and the supernatants were incubated with the indicated antibodies for immunoprecipitation overnight at 4 °C. Next, the mixture of supernatants and antibodies was incubated with Protein A/G PLUS-Agarose (Santa Cruz) for 1 h at 4 °C. The agarose was then collected, washed with lysis buffer at least 4 times, and denatured in loading buffer. The samples were further analyzed by Western blotting with the indicated antibodies.

### Statistical analysis

All the statistical analyses were carried out *via* Prism 10 (GraphPad). All the data are presented as the mean ± standard deviation. One-way analyses of variance and t tests were used for statistical comparisons. *p* values were categorized as follows: ∗*p* < 0.05; ∗∗*p* < 0.01; ∗∗∗*p* < 0.001; and ∗∗∗∗*p* < 0.0001.

## Data availability

The data supporting the findings of this study are available upon reasonable request to the corresponding authors.

## Supporting information

This article contains supporting information.

## Conflict of interest

The authors declare that they have no conflicts of interest with the contents of this article.
