# Supplementary material for: Ubiquitin-specific peptidase 25 ameliorates hepatic steatosis by stabilizing peroxisome proliferator-activated receptor alpha
Source: J Biol Chem. 2024 Oct 11;300(11):107876. doi: 10.1016/j.jbc.2024.107876 (PMC11570943; doi:10.1016/j.jbc.2024.107876)
Supplement: Supporting information [file mmc1.docx]

Ubiquitin-specific peptidase 25 ameliorates hepatic steatosis by stabilizing peroxisome proliferator activated receptor alpha.

Peihao Liu^1, 2, *^, Xin Song^1, *^, Qingxia Chen^1, *^, Li Cen^1, 2^, Chenxi Tang^1^, Chaohui Yu^1, #^, Chengfu Xu^1, #^

1, Department of Gastroenterology, The First Affiliated Hospital, Zhejiang University School of Medicine, Hangzhou 310003, China.

2, Department of Gastroenterology, Affiliated Hangzhou First People’s Hospital, Westlake University School of Medicine, Hangzhou 310058, China Key Laboratory of Integrated Traditional Chinese and Western Medicine for Biliary and Pancreatic Diseases of Zhejiang Province, Hangzhou 310006, China. Hangzhou Hospital & Institute of Digestive Diseases, Hangzhou 310006, China.

*, PL, XS, and QC contributed equally to this work.

#, Corresponding author: CX ([xiaofu@zju.edu.cn](mailto:xiaofu@zju.edu.cn)), CY ([zyyyych@zju.edu.cn](mailto:zyyyych@zju.edu.cn))

Supplementary Figure and Table Legends

Figure S1 Usp25 knockout mouse construction. gRNA target sequences were: GGACCCTGAGATGTACTCGCTGG; CCGTACTGCTTGCATGAGTGAGG.

Figure S2 Usp25 deficiency exacerbated HFD-induced hepatic steatosis. (A) Representative western blot showing that Usp25 was knocked out in the livers of Usp25^-/-^ mice. Fasting body weights (B) and liver weights (C) of wild-type (WT) and Usp25 knockout (Usp25^-/-^) mice fed an SCD for 16 weeks (*n*=5 in each group). (D) Representative H&E staining and oil red O staining of the indicated groups (200× magnification). (E) Representative western blot showing stable USP25 knockdown in Huh7 cell lines (*n*=3 in each group). (F) Representative western blot showing the overexpression of USP25 in Huh7 cell lines (*n*=3 in each group).

Figure S3 The PPAR pathway was enriched in Usp25^-/-^ mice.

Figure S4 Usp25 could modulate Pparα expression. (A) Representative western blot of the indicated protein expression in the indicated groups (*n*=9 in each group) and quantification of the indicated protein level normalized to that of GAPDH. (B) Representative western blot of the indicated proteins in the indicated groups (*n*=4 in the control group, *n*=5 in the AZ1 group) and quantification of the indicated protein level normalized to that of GAPDH. The data are expressed as the mean ± standard deviation (SD) and were analyzed by Student's t test. **P*< 0.05; ***P*< 0.01; ****P*< 0.001.

Figure S5 USP25 interacts with PPARα and stabilizes it through deubiquitination. Western blotting analysis of the ubiquitination of PPARα in HEK293T cells transfected with mutant ubiquitin (K6, K11, K27, K29, or K33) and PPARα. MG132 was added for 6 h before harvest.

Table S1 Primary antibodies for western blotting and immunoprecipitation.

Table S2 Primer sequences for qPCR and shRNA sequences for USP25.

Figure S1


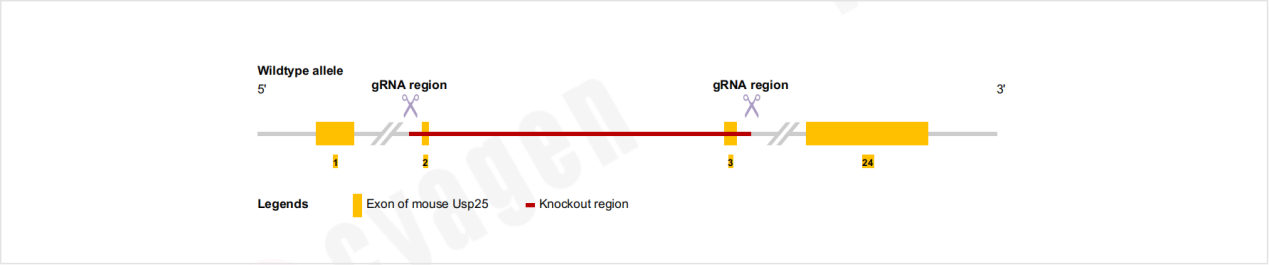


Figure S2


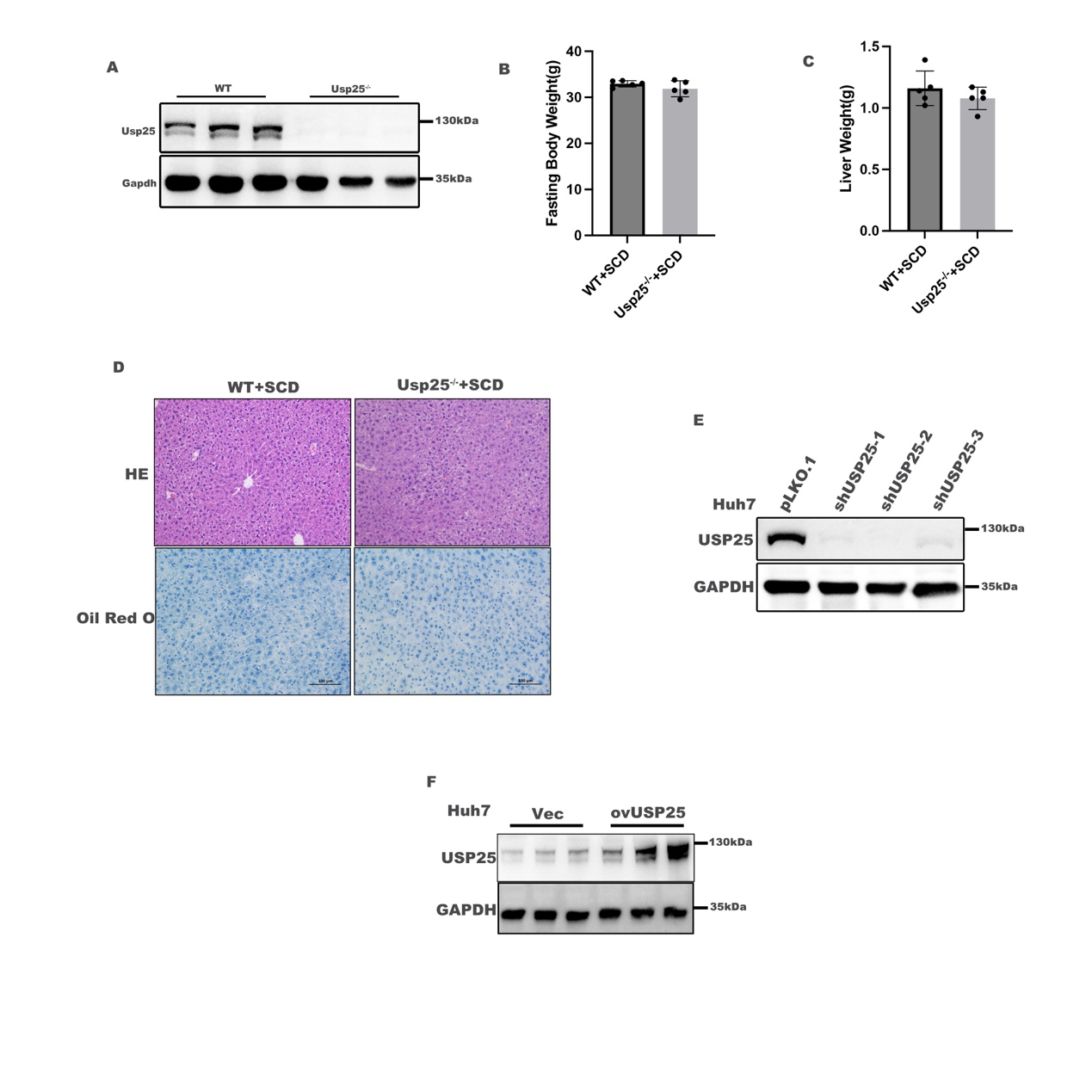


Figure S3


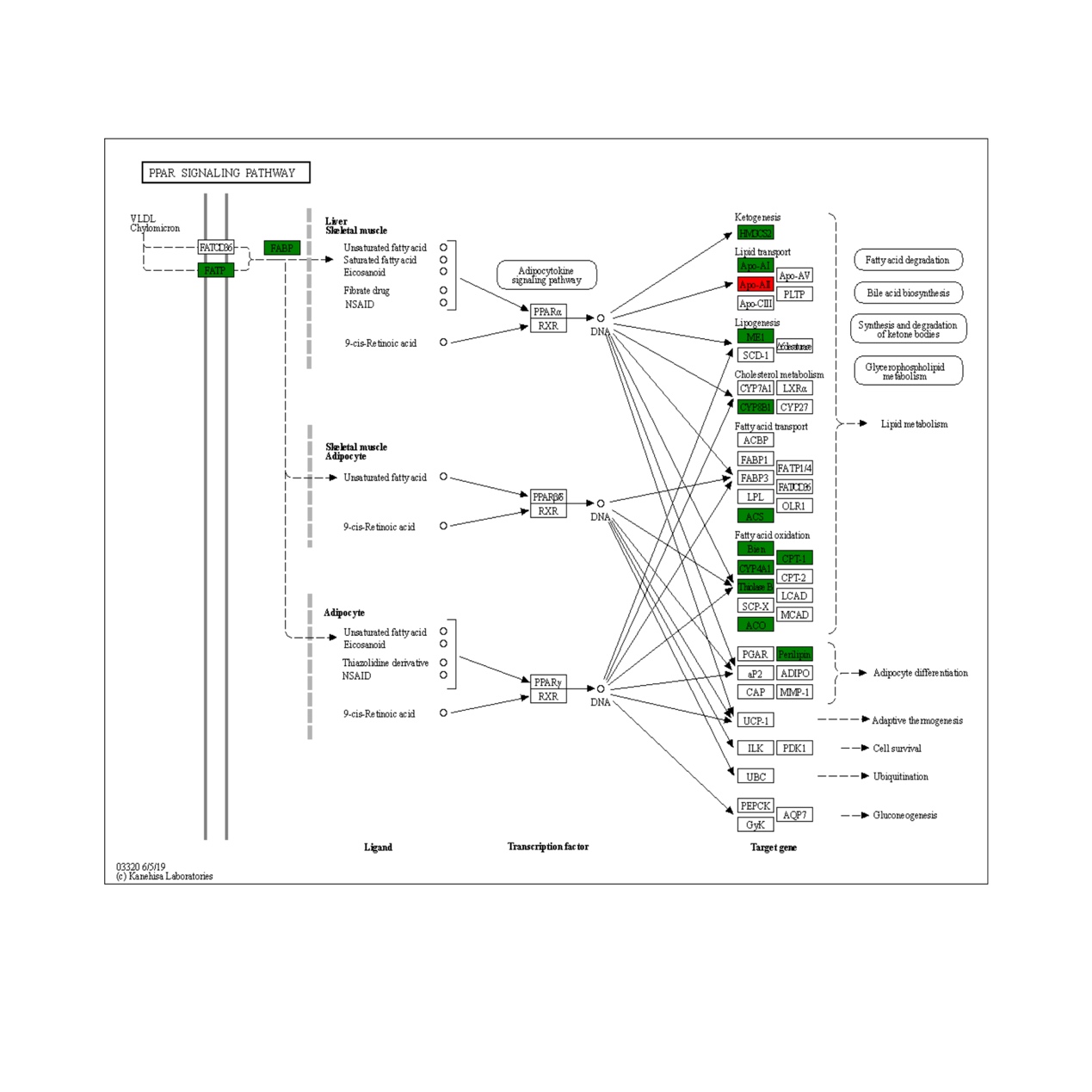


Figure S4


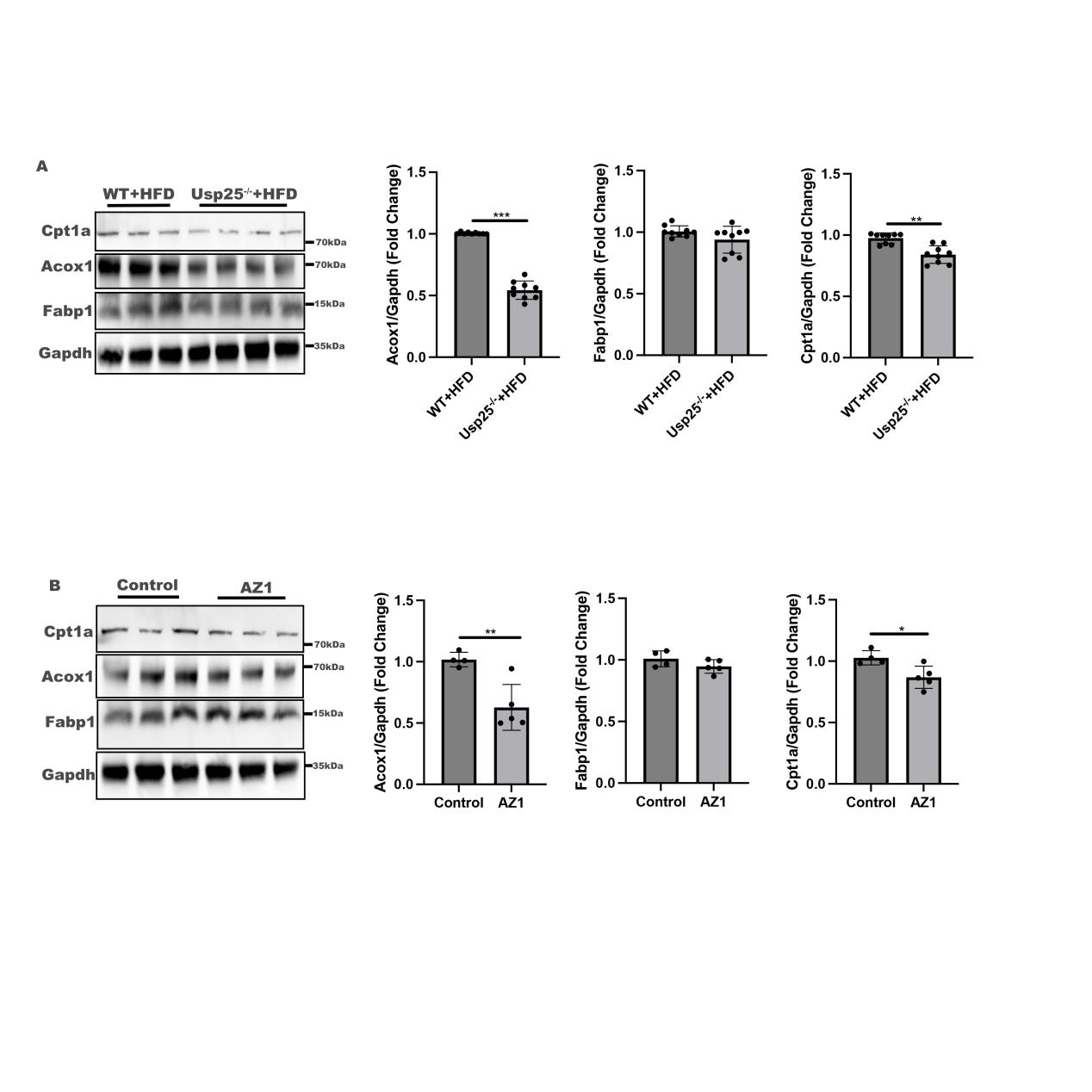


Figure S5


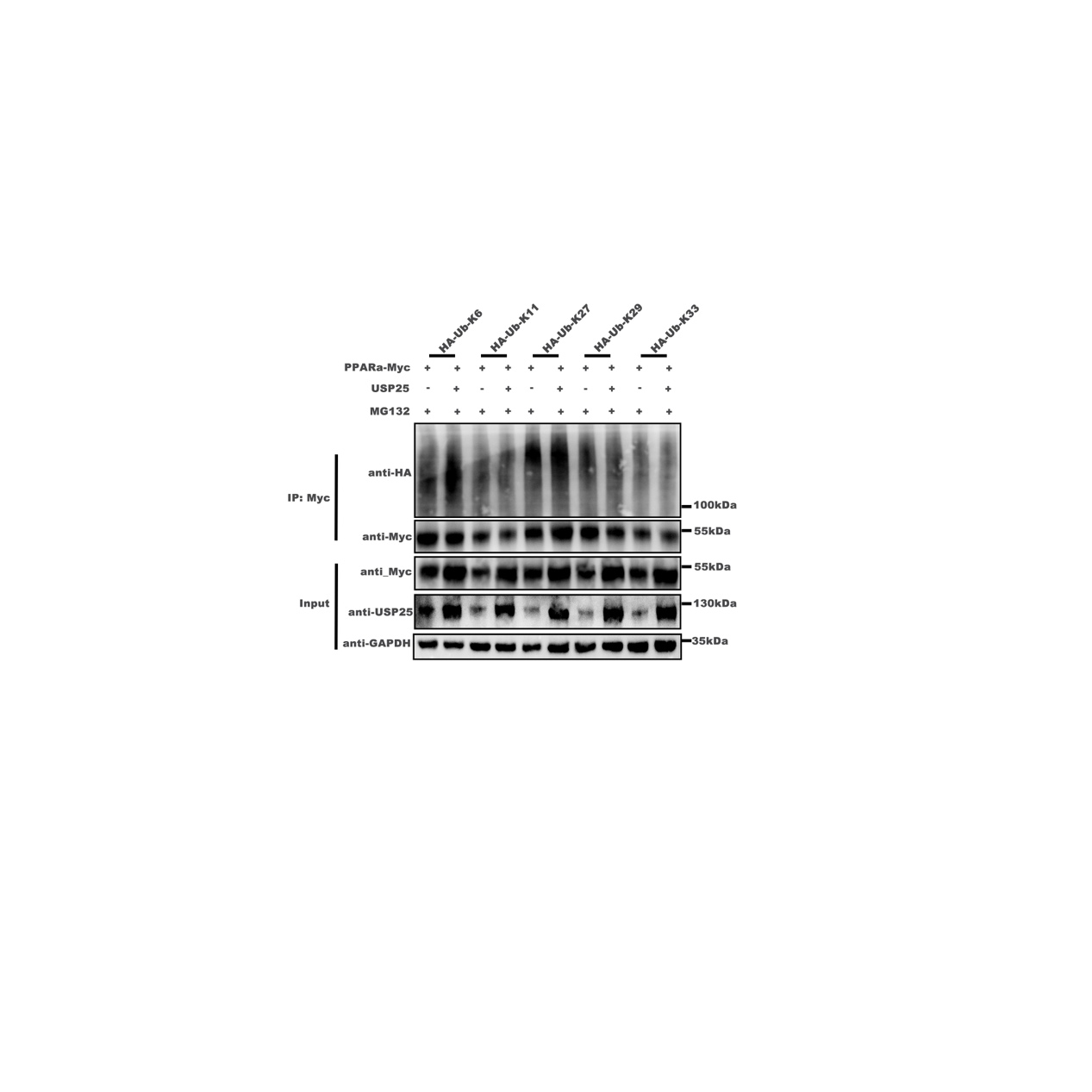


Table S1

| Anti-USP25 | Abcam, ab187156, ab246948 |
| --- | --- |
| Anti-PPARa | Proteintech, 66826-1-Ig |
| Anti-GAPDH | Proteintech, HRP-60004 |
| Anti-Myc | Proteintech, HRP-60003 |
| Anti-HA | Invitrogen, 26183 |
| Anti-HA agarose | Pierce, 26181 |
| Anti-Myc agarose | Pierce, 20169 |
| Anti-Acox1 | Proteintech,10957-1-AP |
| Anti-Cpt1a | Proteintech,66039-1-Ig |
| Anti-Fabp1 | Proteintech,68227-1-Ig |

Table S2

| Gene | Forward | Reverse |
| --- | --- | --- |
| Acox1 | GCTCAGCAGGAGAAATGG | CTCGAGTGATGAGCTGAGC |
| Cpt1a | AGGACCCTGAGGCATCTATT | ATGACCTCCTGGCATTCTCC |
| Fabp1 | TGGTCCGCAATGAGTTCACCCT | CCAGCTTGACGACTGCCTTGACTT |
| Ppara | GAGAATCCACGAAGCCTACC | GCCTCTTTGTCTTCGACGC |
| Gapdh | TGGCCTTCCGTGTTCCTAC | GAGTTGCTGTTGAAGTCGCA |
| shUSP25-1 | TGGAGGAGTAAGATGAAATAT |  |
| shUSP25-2 | GCGTGAGCTGAGGTATCTATT |  |
| shUSP25-3 | GCTGTAGAAGATATGAGAAAT |  |
